# The 79th Annual Meeting of the European Board of Ophthalmology, alongside the UEMS Meeting

**DOI:** 10.22336/rjo.2024.39

**Published:** 2024

**Authors:** Cornel Ştefan, Marian Burcea, Alina Gheorghe

Dear colleagues, we are delighted to share the key topics discussed and agreed upon at the 79th edition of the General Assembly of the European Board of Ophthalmology (EBO) in conjunction with the annual UEMS meeting, held this year in Strasbourg, France.

The Annual Meeting of the European Board of Ophthalmology is a significant event within the European ophthalmology community. It brings together experts, clinicians, researchers, and professionals in the field of ophthalmology to discuss the latest innovations, research, and practices in treating ocular diseases, and to engage in discussions on curricula and training programs for young ophthalmologists.

The event typically includes sessions for scientific paper presentations, hands-on workshops, ongoing professional development courses, and discussions on key topics of interest in ophthalmology. Moreover, the meeting serves as a platform for exchanging knowledge between ophthalmologists from different countries and establishing partnerships in international research projects.

More than 40 members participated in the EBO meeting this year, while over 50 attended the UEMS meeting. These sessions are often held together to facilitate the exchange of ideas and discussions. This year, the main focus was on harmonizing ophthalmology education and training programs throughout Europe.

A key highlight of the meeting was the EBO exam, known as the “European Board of Ophthalmology Diploma” (EBOD), which represents a standardized examination at the European level for ophthalmologists who wish to certify their competencies at an internationally recognized level. The EBOD diploma is widely accepted and recognized throughout Europe. Successful candidates are awarded the title of Fellow of the European Board of Ophthalmology (FEBO), a prestigious and globally acknowledged distinction.

The exam for obtaining the European Board of Ophthalmology Diploma (EBOD) is an important step for ophthalmologists. Recently, some significant updates and changes related to this exam have been made: Increased Emphasis on Clinical Experience, while candidates are now being required to show that they have accumulated a sufficient number of practice years and have encountered a broad spectrum of clinical cases. New Examination Topics: New subjects have been added to reflect recent advancements in ophthalmology, including topics related to new technologies, cutting-edge treatments, and research discoveries. Updated Exam Standards: Examiners are trained to ensure that questions and clinical cases reflect the most recent practice standards and the knowledge required for modern ophthalmologists. EBO has expanded the resources available for candidates by providing detailed guidelines, sample questions, and other educational materials to aid in exam preparation. National and regional initiatives for preparatory courses have also been promoted, aiming to support candidates in training for the exam.

The EBOD exam still consists of two main parts: a written and an oral test. The written part contains multiple-choice questions (MCQs), while the oral part includes discussions based on clinical cases. Many Romanian residents in their final year or young specialists participate in and pass the EBOD exam.

Romania also offers two EBO-accredited training centers for young residents coming from anywhere in Europe. Represented by the Ophthalmology Department at “Dr. Carol Davila” Military Hospital in Bucharest and the Bucharest Clinical Emergency Hospital for Ophthalmology, the centers host residents for a month. Applications can be submitted through the official website (http://www.ebo-online.org), under the Residents Exchange Centers section.

The end of the meeting came with an announcement of great joy and honor for us: the 80th meeting, an anniversary event, will take place in Bucharest on May 30-31, 2025. We invite everyone to come up with ideas and proposals to enhance our education and performance, thereby increasing satisfaction for ourselves and, especially, our patients.

